# Vital staining for cell death identifies Atg9a-dependent necrosis in developmental bone formation in mouse

**DOI:** 10.1038/ncomms13391

**Published:** 2016-11-04

**Authors:** Yusuke Imagawa, Tatsuya Saitoh, Yoshihide Tsujimoto

**Affiliations:** 1 Department of Molecular and Cellular Biology, Research Institute of Osaka Medical Center for Cancer and Cardiovascular Diseases, 1-3-2 Nakamichi, Higashinari-ku, Osaka 537-8511, Japan; 2 Laboratory of Molecular Genetics, Department of Medical Genetics, Graduate School of Medicine, Osaka University, 2-2 Yamadaoka, Suita, Osaka 565–0871, Japan; 3 Department of Inflammation Biology, Institute for Enzyme Research, Tokushima University, 3-18-15 Kuramoto-cho, Tokushima 770-8503, Japan; 4 Laboratory of Host Defense, WPI Immunology Frontier Research Center, Osaka University, 3-1 Yamadaoka, Suita, Osaka 565-0871, Japan

## Abstract

Programmed cell death has a crucial role in various biological events, including developmental morphogenesis. Recent evidence indicates that necrosis contributes to programmed cell death in addition to apoptosis, but it is unclear whether necrosis acts as a compensatory mechanism for failure of apoptosis or has an intrinsic role during development. In contrast to apoptosis, there have been no techniques for imaging physiological necrosis *in vivo*. Here we employ vital staining using propidium iodide to identify cells with plasma membrane disruption (necrotic cells) in mouse embryos. We discover a form of necrosis at the bone surface, which does not occur in embryos with deficiency of the autophagy-related gene *Atg9a*, although it is unaffected by *Atg5* knockout. We also find abnormalities of the bone surface in *Atg9a* knockout mice, suggesting an important role of Atg9a-dependent necrosis in bone surface formation. These findings suggest that necrosis has an active role in developmental morphogenesis.

## Abstract (web summary)

Apoptosis occurs in numerous developmental processes but a role for necrosis in development is unclear. Here, the authors develop a detecting system of necrosis in the developing mouse and find a form of necrosis that is dependent on the autophagy-related gene *Atg9a*.

## Graphical abstract

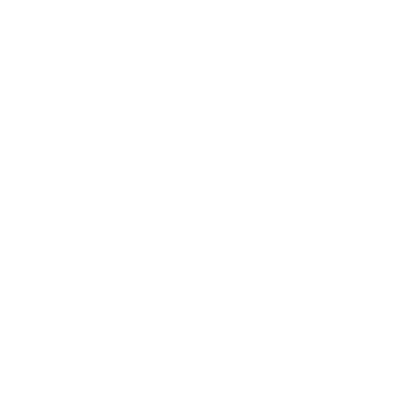

In metazoans, programmed cell death^1^ is involved in various biological processes, including morphogenesis during development, cell turnover and elimination of harmful cells. It is generally considered that programmed cell death is mainly mediated by apoptosis. However, it has been reported that cells displaying morphological characteristics of non-apoptotic death can also be observed at sites where programmed cell death occurs^2^,^3^,^4^,^5^. Based only on morphological features, developmental programmed cell death has been categorized into type 1 (apoptosis), type 2 (autophagic degeneration), type 3A (non-lysosomal disintegration) and type 3B (‘cytoplasmic' degeneration)^6^,^7^. Although apoptosis has been extensively analysed during the past decade, type 2 and type 3 programmed cell death, which are considered to be forms of necrotic death, have not attracted as much attention. Concerning type 2, it has not been determined whether autophagy is activated for cell death or cell survival. Recently, molecular approaches have been employed to analyse some forms of non-apoptotic programmed cell death in animals^8^. For example, it has been reported linker cells that locate between the gonad and cloacal tube undergo non-apoptotic programmed death during development of *Caenorhabditis elegans*, with this form of death being independent of caspase and other apoptotic genes and morphologically resembling necrosis^3^. In *Drosophila melanogaster*, it was reported that nurse cells die by a non-apoptotic mechanism during oocyte maturation, although the morphologic features of the dying cells were not been described^9^, and that death of salivary gland cells occurs through a mechanism regulated by autophagy^10^. In mammals, it has been reported that mammary gland regression after lactation in mice is mediated by necrotic cell death involving the induction of lysosomal enzymes^4^. Furthermore, studies of cultured cells have elucidated the mechanisms of several forms of necrotic programmed cell death, including autophagy-dependent cell death^11^,^12^, necroptosis (programmed necrosis)^13^ and ferroptosis^14^. Autophagy-dependent death is a form of cell death that is suppressed by inhibition of autophagy, and might underlie type 2 programmed cell death. Necroptosis is a type of necrotic cell death that depends on receptor-interacting serine/threonine-protein kinase 1 (RIP1) and RIP3, as well as mixed lineage kinase domain-like protein (MLKL)^15^,^16^,^17^,^18^,^19^. Although necroptosis has been implicated in cell death after infection by certain viruses^17^ and has also been linked to several pathological conditions associated with inflammation^20^, it has not been determined whether necroptosis is involved in physiological programmed cell death. Taken together, these results suggest that multiple mechanisms of cell death operate in animals, with both apoptosis and necrosis contributing to programmed cell death.

Mutant mice with deficiency of key apoptotic factors, such as *Apaf-1* knockout (KO) mice, *caspase-9* KO mice, and *Bax*/*Bak* double KO mice, show certain morphological abnormalities. For example, *Apaf-1* KO and *caspase-9* KO mice develop exencephaly, especially animals with a 129 background but not a B6 background^21^,^22^,^23^, while *Bax*/*Bak* double KO mice with a B6 background have interdigital webs^24^. These morphological abnormalities are considered to provide evidence that apoptosis has an important role in developmental cell death *in vivo*. However, the defects created by disturbance of apoptosis are limited, and non-apoptotic cell death has been observed in these apoptosis-deficient mice, suggesting the possible activation of compensatory cell death mechanisms that are usually silent. Another possibility is that certain programmed cell death paradigms are intrinsically mediated by non-apoptotic death mechanisms. Thus, it is important to determine whether non-apoptotic cell death is merely a compensatory mechanism for failure of apoptosis or is an intrinsic active process during mammalian development.

We develop a vital staining method that allowed us to distinguish between apoptotic and non-apoptotic (necrotic) cells in mouse embryos. Using this method and apoptosis-deficient mice as well as other mutant mice, we demonstrate that non-apoptotic death makes an intrinsic contribution to programmed cell death during murine embryogenesis in addition to apoptosis.

## Results

### Vital-staining of dead cells *in vivo*

**Figure 1:**
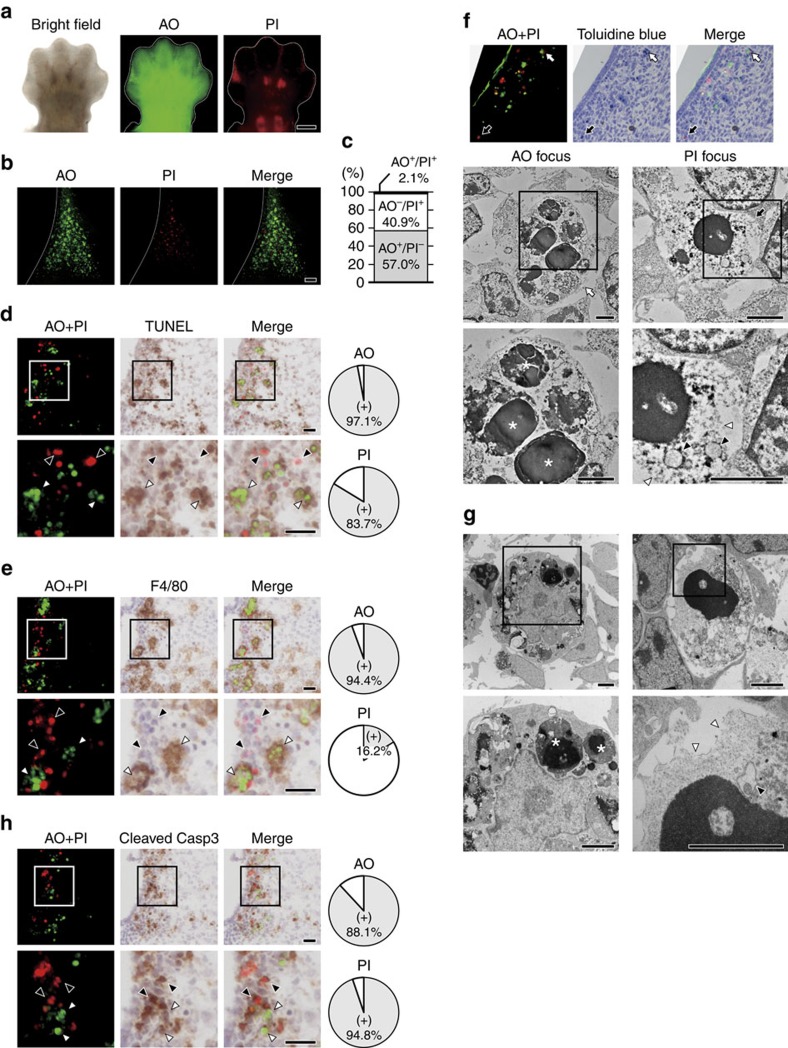
Vital staining of dead cells with AO and PI. (**a**) AO and PI foci in limb buds. The right forelimb bud of an E13.5 embryo injected with AO and PI were visualized by fluorescence stereomicroscopy. Scale bar, 400 μm. (**b**) The first interdigital region of the forelimb bud of an E13.5 embryo injected with AO and PI was observed by laser scanning confocal microscopy, and maximum intensity projection images were constructed. Scale bar, 50 μm. (**c**) Most of the PI foci and AO foci did not overlap. AO and PI foci in the first interdigital region of the forelimb bud of an E13.5 embryo were separated from background signals by the threshold algorithm ‘Intermodes'^31^ and were counted in 15 tissue sections. (**d**) A high proportion of the AO-positive cells and PI-positive cells were also TUNEL-positive. Axial cryosections of the first interdigital region of the forelimb bud from an E13.5 embryo injected with AO and PI were subjected to TUNEL staining. White arrowheads and black arrowheads indicate AO and PI foci, respectively. The pie chart shows the TUNEL-positive rates of AO foci and PI foci. Scale bar, 20 μm. (**e**) The majority of AO-positive cells, but not PI-positive cells, were engulfed by macrophages. Cryosections of the first interdigital region of the forelimb bud from an E13.5 embryo were subjected to immunostaining with an anti-F4/80 antibody. (**f**) AO-positive cells showed evidence of phagocytosis, while PI-positive cells showed necrotic changes. TEM examination of an AO-positive cell (white arrow) and a PI-positive cell (black arrow). * apoptotic cell or apoptotic body, black arrowhead: swollen organelle, white arrowhead: ruptured plasma membrane. Scale bar, 2.5 μm. (**g**) Dead cells with similar morphologic features to AO-positive cells and PI-positive cells were also observed without vital staining. After euthanasia, an E13.5 embryo without AO or PI injection was rapidly fixed and examined by TEM. (**h**) The majority of AO-positive cells and PI-positive cells were also positive for cleaved caspase-3. Cryosections of the first interdigital region of the forelimb bud from an E13.5 embryo were subjected to immunostaining with an anti-cleaved caspase-3 antibody.

**Figure 2:**
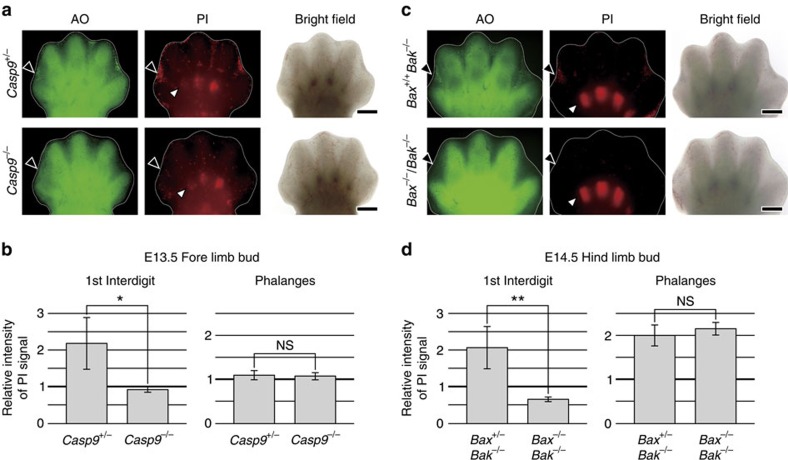
AO and PI foci in apoptosis-deficient mice. (**a**) Lack of AO and PI foci in the interdigital regions of *caspase-9* KO embryos. At E13.5, a *caspase-9* KO (*Casp9*^−/−^) embryo and a control (*Casp9*^+/−^) embryo were injected with AO and PI, after which the right forelimb buds were observed by fluorescence stereomicroscopy. The first interdigital regions and phalanges are indicated by black and white arrowheads, respectively. Scale bar, 400 μm. (**b**) Bar graphs show quantitative assessment of PI signals in the first interdigital region and phalanges. Left: The relative intensity of PI signals in the first interdigital region was calculated as the PI signals in the region divided by background PI signals in the second digit (see Supplementary Fig. 1a). Data are represented as the mean ±s.d.; *n*=9 for *Casp9*^+/−^ embryos and *n*=3 for *Casp9*^−/−^ embryos. *P= 0.014 for comparison between *Casp9*^−/−^ and *Casp9*^+/−^ embryos by the two-tailed Student's *t*-test. Right: The relative intensity of PI signals in the phalangeal area was calculated as the PI signals in that area divided by the PI signals in the palmar region (see Supplementary Fig. 1b). Data are represented as the mean ±s.d.; *n*=9 for *Casp9*^+/−^ embryos and *n*=3 for *Casp9*^−/−^ embryos. NS: *P*=0.776 for comparison between *Casp9*^−/−^ and *Casp9*^+/−^ embryos. Note that PI signals in the phalangeal area were weaker at E13.5 than at E14.5. (**c**). Lack of AO and PI foci in the interdigital regions of *Bax*/*Bak* double KO embryos. At E14.5, a *Bax*/*Bak* double KO (*Bax*^−/−^/*Bak*^−/−^) embryo and a control (*Bax*^+/+^/*Bak*^−/−^) embryo were injected with AO and PI, and the right hind limb buds were observed. (**d**) Bar graphs show quantitative assessment of PI signals in the first interdigital region and the phalanges. Data are represented as the mean ±s.d.; *n*=6 for *Bax*^+/−^/*Bak*^−/−^ embryos and *n*=4 for *Bax*^−/−^/*Bak*^−/−^ embryos. **P=0.0014, NS: *P*=0.288 for comparison between *Bax*^−/−^/*Bak*^−/−^ and *Bax*^+/−^/*Bak*^−/−^ embryos by the two-tailed Student's *t*-test.

To determine whether or not non-apoptotic cell death has an intrinsic role in developmental programmed cell death in embryonic mice, we employed vital staining to differentiate apoptotic cells and necrotic cells. Acridine orange (AO) is a membrane-permeable cationic dye that stains living cells, and is also widely used for *in vivo* staining of apoptotic cells that have been engulfed by phagocytes without disruption of the plasma membrane^25^. Engulfed apoptotic cells show stronger AO signals than living cells, suggesting that AO can be used to monitor phagolysosomal activity after engulfment of apoptotic cells by phagocytes. A common feature of necrotic death is disruption of the plasma membrane^26^,^27^. Therefore, we reasoned that the membrane-impermeable dye propidium iodide (PI) could be used for *in vivo* staining of necrotic cells. To verify the feasibility of employing this vital staining with AO and PI to identify apoptotic cells and necrotic cells, respectively, we injected these dyes into the yolk sac veins of mouse embryos *ex vivo* since little PI crosses the placenta. As shown in Fig. 1a,b, strongly positive AO dots were observed in the interdigital region of the forelimb bud in E13.5 embryos, which is known as a site of regression involving apoptosis^28^,^29^,^30^. While AO also weakly stained viable cells throughout the forelimb bud, the stronger AO signals in the interdigital region could be separated from weak signals by using the threshold algorithm ‘Intermodes'^31^ in the tissue sections. In addition to AO-positive cells that were presumably apoptotic cells, we also unexpectedly identified PI-positive cells (presumably necrotic cells) in the interdigital region of the forelimb bud (Fig. 1a,b). Most of the PI signals and AO signals did not overlap (Fig. 1c). It has been reported that separation of the digits occurs at E13–E14 in the forelimb buds and at E14–E15 in the hind limb buds^32^. In agreement with this report, we observed similar findings in hind limb buds at the slightly later stage of E14.5 (refer to Figs 2 or 4 [Fig f3][Fig f4]). Then we performed the TdT-mediated dUTP nick-end labelling (TUNEL) assay on AO- and PI-labelled cells to detect double-stranded DNA breaks as an indicator of cell death. While apoptotic cells are strongly TUNEL-positive, it has been reported that even necrotic cells can be labelled if double-stranded DNA breaks occur^33^. As shown in Fig. 1d, the majority of the AO-positive cells and PI-positive cells were also TUNEL positive, indicating that both AO-positive cells and PI-positive cells contained double-stranded DNA breaks. Next, we employed immunohistochemistry (IHC) with an antibody for F4/80 (a macrophage marker) to investigate whether the AO- or PI-positive cells had been engulfed by macrophages. We found that almost all of the AO-positive cells were surrounded by positive signals for F4/80, while PI-positive cells were only occasionally stained by the anti-F4/80 antibody (Fig. 1e), and nearly half of the F4/80-stained PI-positive cells were also AO-positive. We also performed transmission electron microscopy (TEM) to allow direct ultrastructural observation of the AO- and PI-positive cells. Consistent with the results of TUNEL staining and IHC using anti-F4/80 antibody, TEM revealed that the AO-positive cells were apoptotic cells or bodies engulfed by macrophage (Fig. 1f) as observed by Kerr *et al*.^34^, while PI-positive cell displayed necrotic characteristics such as disruption of the plasma membrane, swelling of organelles and condensation of chromatin (Fig. 1f). Dead cells with the same morphological characteristics as AO-positive cells or PI-positive cells were observed in embryos, which were immediately fixed after dissected without vital staining (Fig. 1g). These findings suggested that AO staining identified apoptotic cells after phagocytosis by macrophages without disruption of the plasma membrane, while PI staining identified necrotic cells with plasma membrane disruption. Although it has been thought that cells of the interdigital region mainly undergo regression via apoptosis in mice, we observed both AO-positive apoptotic cells and PI-positive necrotic cells in this region (Fig. 1a–g). Both the AO-positive cells and the PI-positive cells were stained by IHC using anti-cleaved caspase-3 antibody, indicating activation of apoptosis (Fig. 1h). Moreover, neither AO-positive cells nor PI-positive cells were observed in *caspase-9* KO mice and *Bax*/*Bak* double KO mice, which are deficient in essential genes for apoptosis (Fig. 2a,c; black arrowhead, b and d; left graph, and Supplementary Fig. 1a), indicating that both the AO-positive apoptotic cells and PI-positive necrotic cells were initially generated by activation of apoptotic machinery. This suggested that PI-positive necrotic cells in the interdigital region were probably cells at a late stage of apoptosis, which had undergone secondary necrosis with plasma membrane disruption.

### Intrinsic necrosis during normal development

**Figure 3 f3:**
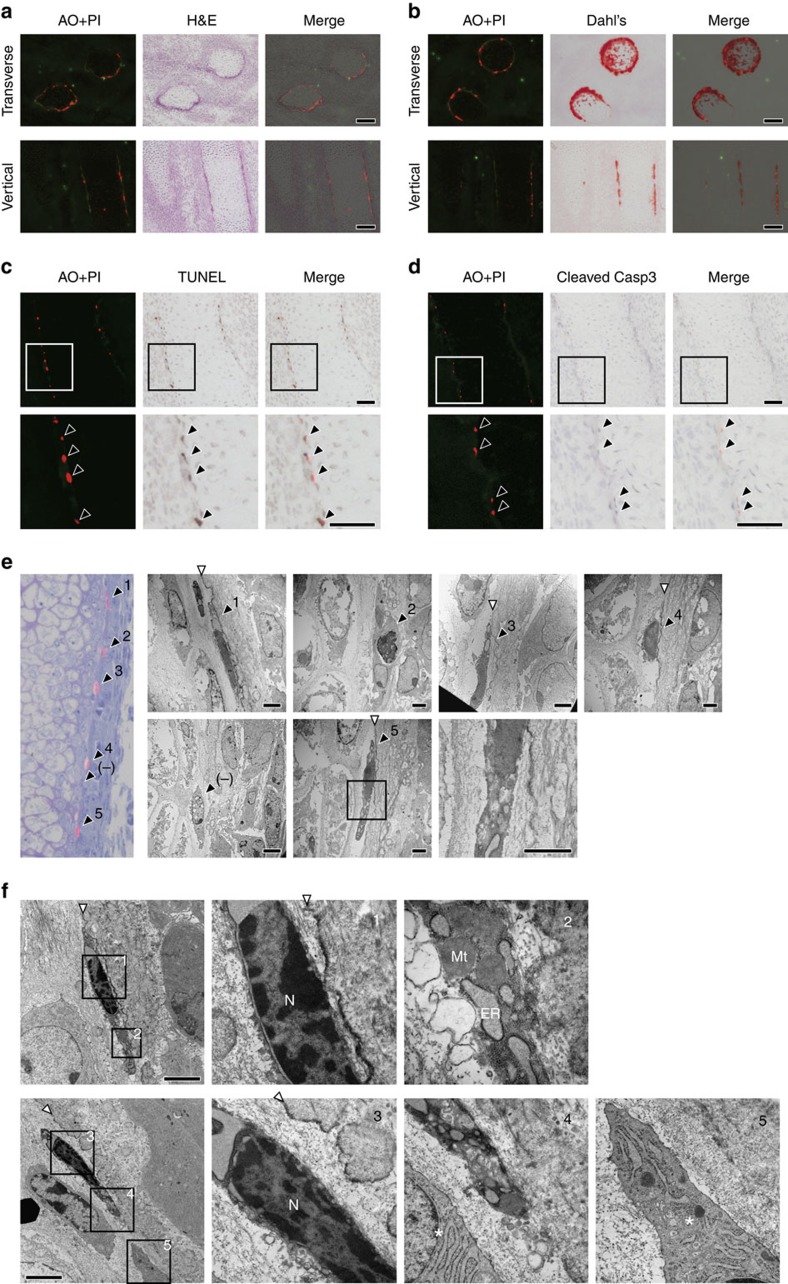
Necrotic morphology of PI-positive cells at the bone surface. (**a**) PI foci at the bone surface. Cryosections of the forelimb zeugopod of an E14.5 embryo injected with AO and PI were observed by fluorescence microscopy, and then the sections were stained by haematoxylin and eosin (H&E). Scale bar, 100 μm. (**b**) PI foci at sites of calcification (positive for Dahl's calcium stain). Cryosections of the forelimb zeugopod of an E14.5 embryo injected with AO and PI were observed by fluorescence microscopy, followed by staining with Alizarin Red S. (**c**) PI-positive cells are also positive for TUNEL staining. A vertical cryosection of the forelimb of an E14.5 embryo injected with AO and PI was observed by fluorescence microscopy and then TUNEL staining was performed. Black arrowheads indicate PI foci. Scale bar, 50 μm. (**d**) PI-positive cells are negative for cleaved caspase-3. A vertical cryosection of the forelimb of an E14.5 embryo injected with AO and PI was subjected to immunostaining by an anti-cleaved caspase-3 antibody. (**e**) PI-positive cells show necrotic morphology. PI-positive cells were examined by TEM as described in Methods. Numbers on the electron micrographs correspond to the numbers of the PI-positive cells shown in the left panel. (−) indicates a PI-negative cell. White arrowheads indicate the lamina limitans. Scale bar, 2 μm. (**f**) Dead cells exhibiting similar morphology to PI-positive cells in an embryo without AO and PI staining. After euthanasia, an E14.5 embryo without injection of AO and PI was rapidly fixed and examined by TEM. The numbered panels are magnifications of the respective regions enclosed by squares on the left micrographs. Cells indicated by asterisks in panels 4 and 5 are normal cells with an intact plasma membrane. White arrowheads indicate the lamina limitans. ER, endoplasmic reticulum, N, nucleus, Mt, mitochondria.

Even under apoptosis-deficient conditions, PI-positive cells were still detected at sites other than the interdigital region in the autopod of the limb bud (Fig. 2a,c; white arrowhead, b and d; right graph, and Supplementary Fig. 1b), implying that these PI foci in the autopod were independent of apoptosis. The PI foci seemed to be confined to sites of bone morphogenesis. Consistent with this concept, two groups of PI foci were also observed in the forelimb zeugopod at sites corresponding to the ulna and radius (Fig. 1a). To confirm the notion that necrotic cell death was occurring at sites of bone morphogenesis, we performed detailed analysis of PI foci in the zeugopod, where these foci formed two cylindrical groups (Fig. 3a). Using Dahl's calcium staining to stain sites of calcification with Alizarin Red S, we found that the PI foci existed at sites where calcification was occurring (Fig. 3b), suggesting that these foci were specifically located at regions of bone surface development. We also performed TUNEL staining and IHC using anti-cleaved caspase-3 antibody. We found that PI-positive cells were positive for TUNEL stain (Fig. 3c), although staining was weaker than in a nearby AO focus (Supplementary Fig. 2a). In addition, PI-positive cells were not stained by IHC using anti-cleaved caspase-3 antibody (Fig. 3d), although an AO-positive cell was stained by this antibody in the same tissue section (Supplementary Fig. 2b). These results suggested that PI foci at the prospective bone surface represented cells undergoing non-apoptotic death. In addition, direct ultrastructural observation of the PI-positive cells by TEM revealed rupture of the plasma membrane and swelling of organelles such as the mitochondria and endoplasmic reticulum (Fig. 3e), which are characteristic morphologic features of necrotic cells^26^,^27^. Moreover, we found that the PI-positive cells were surrounded by collagen fibrils. Interestingly, an electron-dense layer was only observed parallel to the necrotic cells (Fig. 3e; white arrowhead) and was not observed next to a PI-negative cell (Fig. 3e; (−)). This electron-dense layer was thought to be the lamina limitans, which is an organic layer between the mineralized and non-mineralized parts of the bone matrix. As shown in panel no. 2 of Fig. 3e, necrotic cells were also observed without an accompanying lamina limitans, but such cells were very rare and were located on the opposite side of the fibrous layer to the other necrotic cells. These findings raised the possibility that necrotic cell death has a role in mineralization of the bone surface. Similar morphologic features were also observed at the prospective bone surface of an embryo without vital staining (Fig. 3f). Taken together, our data indicate that physiological necrosis occurs at the prospective bone surface during normal bone morphogenesis.

### Crucial role of Atg9a in necrosis during bone morphogenesis

**Figure 4 f4:**
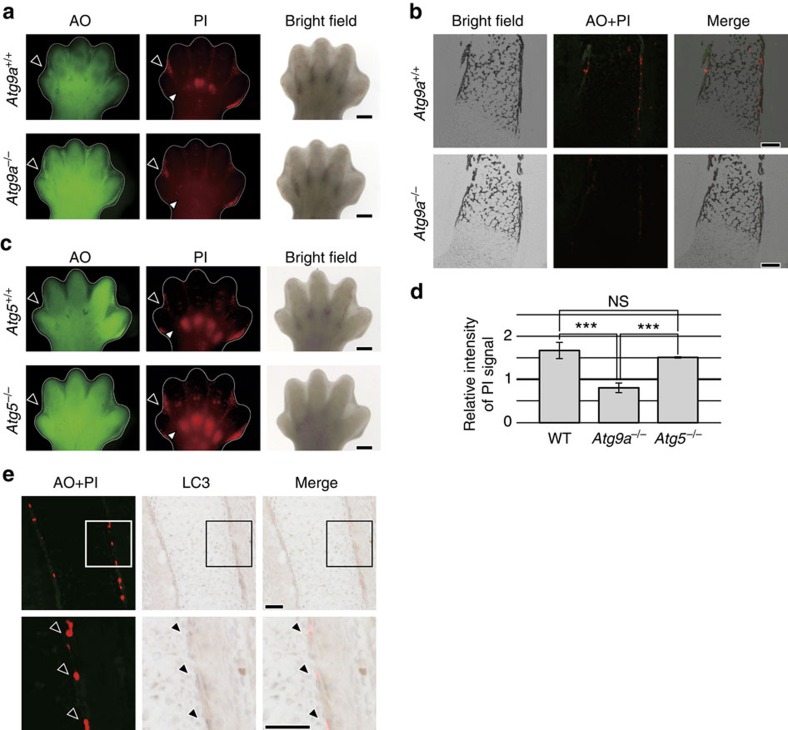
Lack of PI foci at the bone surface in *Atg9a* KO embryos. (**a**) *Atg9a* KO embryos had no PI foci in the phalanges, but these foci persisted in the interdigital regions. At E14.5, an *Atg9a* KO (*Atg9a*^−/−^) embryo and a control (*Atg9a*^+/+^) embryo were injected with AO and PI, after which the right hind limb buds were observed by fluorescence stereomicroscopy. Black and white arrowheads indicate the first interdigital regions and phalangeal areas, respectively. Scale Bar, 400 μm. (**b**) PI foci were not observed in the ulna at a later stage of *Atg9a* KO embryo. Fluorescence microscopy of a vertical cryosection of the forelimb zeugopod of an E15.5 embryo injected with AO and PI. The black foci in bright fields correspond to calcified areas. Scale bar, 100 μm. (**c**) PI foci in the phalanges of an *Atg5* KO embryo. At E14.5, an *Atg5* KO (*Atg5*^−/−^) embryo and a control (*Atg5*^+/+^) embryo were injected with AO and PI, and the right hind limb buds were observed by fluorescence stereomicroscopy. (**d**) Bar graphs showing quantitative assessment of PI signals in the phalanges. The relative intensity of PI signals in the phalangeal area was calculated as the PI signals in the area divided by the PI signals in the palmar region. Data are represented as the mean ±s.d.; *n*=8 for wild-type (WT) embryos, *n*=5 for *Atg9a*^−/−^ embryos and *n*=4 for *Atg5*^−/−^ embryos. ***P=0.0000016 and *P*=0.0000052 for comparison between *Atg9a*^−/−^ embryos and WT embryos or *Atg5*^−/−^ embryos, respectively, by the two-tailed Student's *t*-test. NS: *P*=0.13 for comparison between *Atg5*^−/−^ and WT embryos. (**e**) LC3 punctae were not observed in PI-positive cells. A vertical cryosection of the forelimb of an E14.5 embryo injected with AO and PI was subjected to immunostaining with an anti-LC3 antibody. Scale bar, 50 μm.

To obtain some insight into the molecular mechanism of necrotic programmed cell death at the prospective bone surface, we searched for KO mice in which this type of necrotic cell death did not occur. In mice with deficiency of *Atg9a*, one of the genes related to autophagy^35^,^36^, PI foci were not observed at the prospective bone surface on E14.5, although PI-positive cells were still observed in the interdigital region (Fig. 4a,d). In addition, *Atg9a* KO embryos did not have PI foci in the ulna, even at the later stage of E15.5 (Fig. 4b). Since PI foci were observed from E13.5 in wild-type embryos (Fig. 1a), this result indicated that the absence of PI-positive cells in *Atg9a* KO embryos was not simply due to delayed development. Further support for this idea was provided by the finding that separation of limb digits occurred normally in *Atg9a* KO embryos. In mice with deficiency of *Atg5*, another gene related to autophagy^37^, PI foci did not disappear from the prospective bone surface, in contrast to *Atg9a* KO mice (Fig. 4c,d). Furthermore, induction of LC3 and formation of LC3 punctae, an indicator of autophagy^38^, were not observed in PI-positive cells on the prospective bone surface (Fig. 4e). Additionally, autophagosomes were not observed in PI-positive cells on the prospective bone surface (Fig. 3e,f), while both induction of LC3 and autophagosomes were seen in cells at other sites in the same section (Supplementary Fig. 3). This suggested that occurrence of necrotic cell death at the prospective bone surface requires Atg9a, but is not dependent on autophagy.

**Figure 5:**
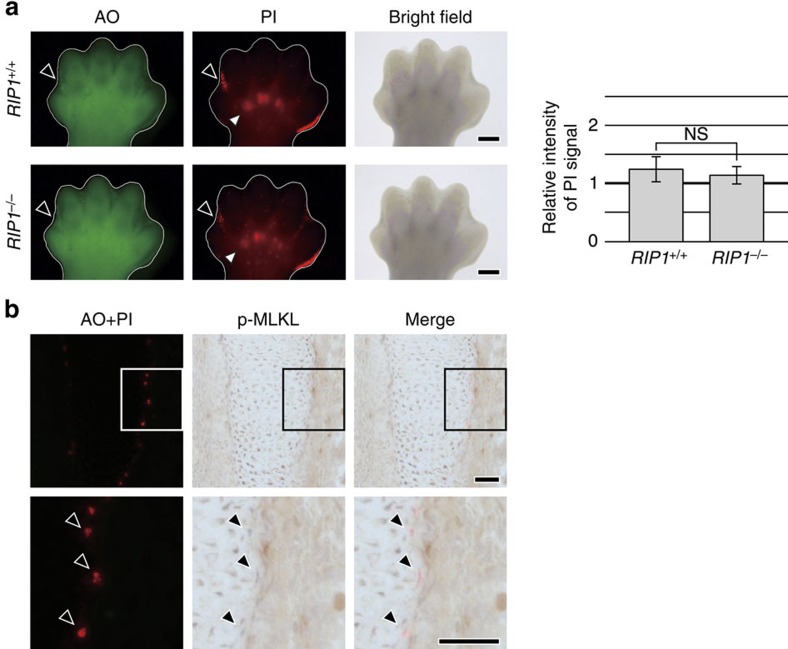
No relation of necroptosis to PI foci at the bone surface. (**a**) PI foci in the phalanges of an *RIP1* KO embryo. At E14.5, an *RIP1* KO (*RIP1*^−/−^) embryo and a control (*RIP1*^+/+^) embryo were injected with AO and PI, and the right hind limb buds were observed by fluorescence stereomicroscopy. Scale bar, 400 μm. Bar graphs show quantitative assessment of PI signals in the phalanges. The relative intensity of PI signals in the phalangeal area was calculated as the PI signals in the area divided by the PI signals in the palmar region. Data are represented as the mean ±s.d.; *n*=3 for *RIP1*^+/+^ embryos and *n*=3 for *RIP1*^−/−^ embryos. NS: *P*=0.158 for comparison between *RIP1*^−/−^ and *RIP1*^+/+^ embryos. Because these embryos were at a slightly earlier stage of development, PI signals were lower in the phalangeal region compared with the other embryos. (**b**) PI-positive cells did not show phosphorylation of MLKL. A vertical cryosection of the forelimb of an E14.5 embryo injected with AO and PI was subjected to immunostaining by an anti-phospho MLKL antibody. Scale bar, 50 μm.

Next, we tried to obtain information about the relationship of Atg9a-dependent cell death to known forms of necrosis. Two forms of necrotic cell death, necroptosis and ferroptosis, are currently receiving considerable attention. Since KO mice and specific activation markers are available for necroptosis, but not for ferroptosis, we assessed the relationship between necroptosis and Atg9a-dependent cell death. In mice with deficiency of RIP1, one of the key molecules involved in necroptosis^15^, PI foci were still observed at the prospective bone surface (Fig. 5a). Phosphorylation of MLKL, an indicator of necroptosis^19^, was not observed in the PI foci on the prospective bone surface (Fig. 5b), although MLKL phosphorylation was detected other sites in the same section (Supplementary Fig. 4). These results suggested that necrotic cell death at the prospective bone surface is not related to necroptosis.

### Bone abnormalities in *Atg9a* KO mice

**Figure 6:**
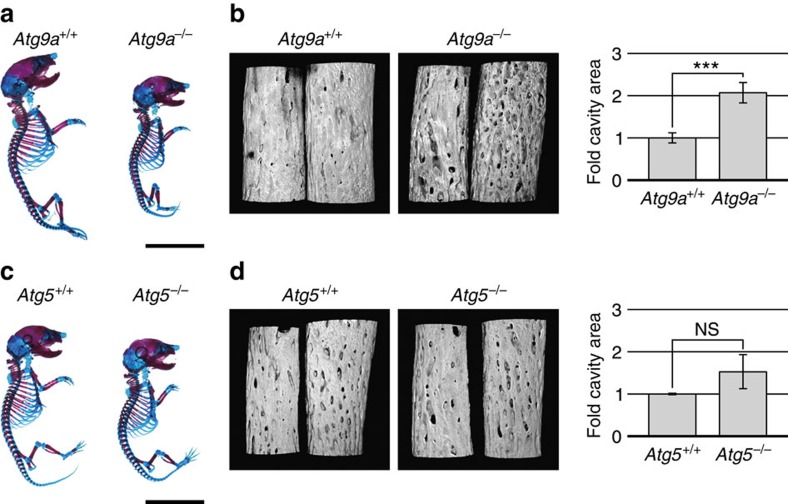
Bone morphology in *Atg9a* KO mice. (**a**) Newborn *Atg9a* KO mice were smaller than newborn wild-type mice, but did not display obvious skeletal dysplasia. Transparent specimens of *Atg9a* KO (*Atg9a*^−/−^) and control (*Atg9a*^+/+^) newborn pups were stained by Alcian Blue and Alizarin Red S. Scale bar, 10 mm. (**b**) Porosity of the bone surface in *Atg9a* KO mice. Forelimb zeugopods of a newborn *Atg9a* KO (*Atg9a*^−/−^) pup and a control (*Atg9a*^+/+^) pup were examined by laser scanning confocal microscopy using Alizarin Red S fluorescence, followed by three-dimensional-rendering of the z-stack images. Bar graphs display quantification of the cavity area in the forelimb zeugopods of *Atg9a*^−/−^ and *Atg9a*^+/+^ pups (as fold changes). Because there was a significant difference of bone morphogenesis between pups from different mothers, we normalized the cavity area ratio by that of wild-type littermates (the mean cavity area in wild-type littermates was set as 1). Data are represented as the mean ±s.d.; *n*=5 for *Atg9a*^+/+^ pups and *n*=3 for *Atg9a*^−/−^ pups. ***P=0.0001 for comparison between *Atg9a*^−/−^ and *Atg9a*^+/+^ pups by the two-tailed Student's *t*-test. (**c**) Transparent specimens of a newborn *Atg5* KO (*Atg5*^−/−^) pup and a control (*Atg5*^+/+^) pup. Scale bar, 10 mm. (**d**) In the *Atg5* KO pup, the bone surface does not display remarkable changes compared with that of the control pup. The same analysis as in **b** was performed for the forelimb zeugopods of newborn *Atg5* KO (*Atg5*^−/−^) pups and control (*Atg5*^+/+^) pups. *n*=3 for *Atg5*^+/+^ pups and *n*=5 for *Atg5*^−/−^ pups. NS: *P*=0.07 for comparison between *Atg5*^−/−^ and *Atg5*^+/+^ pups by the two-tailed Student's *t*-test. There was no significant difference between *Atg5*^−/−^ and *Atg5*^+/+^ pups.

We then investigated whether Atg9a-dependent necrotic programmed cell death has an important physiological role in developmental bone morphogenesis. As reported previously, *Atg9a* KO mice die within one day of delivery like mice deficient for other autophagy genes^36^. Therefore, we compared bone morphology among newborn *Atg9a* KO mice, *Atg5* KO mice and wild-type mice. While *Atg9a* KO mice did not display obvious skeletal dysplasia, they were significantly smaller than newborn wild-type mice and *Atg5* KO mice (Fig. 6a,c). In addition, the bones of *Atg9a* KO mice were more porous than those of wild-type mice or *Atg5* KO mice. Therefore, we performed careful observation of bone surface morphology by laser scanning confocal microscopy of transparent specimens stained with Alizarin Red S. Consistent with our concept that PI-positive cells were localized at the prospective bone surface with lamina limitans, the bone surface was rougher and more porous in *Atg9a* KO mice compared with wild-type mice or *Atg5* KO mice (Fig. 6b,d, and Supplementary Fig. 5). These results suggested that Atg9a-dependent necrosis has an important role during developmental bone morphogenesis in mice.

## Discussion

In this study, we developed a vital staining method that can distinguish between apoptotic and non-apoptotic (necrotic) cells in mouse embryos. It has already been reported that AO labels apoptotic cells both *in vitro* and *in vivo*^25^,^39^,^40^, but the underlying mechanism has not been well documented. From the reports of other groups and our current data, the most convincing explanation is that a plasma membrane-permeable cationic dye such as AO labels phagocytosed apoptotic cells *in vivo* after being incorporated into phagocytes via the H^+^ pump during acidification^25^. On the other hand, a plasma membrane-impermeable nuclear staining dye such as PI labels nuclei after rupture of the plasma membrane. Although PI is widely employed for *in vitro* analysis of cell death by methods such as flow cytometry, there have been very few reports of its use for *in vivo* assessment of cell death^41^. One of the important points of our vital staining method was injection of PI into the yolk sac vein, since we found that merely immersing mouse embryos in PI solution did not work. It is possible that embryonic vessels are relatively leaky so that PI diffuses widely through the tissues of the embryo.

We observed mouse embryos at different stages using this vital staining method. We initially focused on the interdigital region because regression of interdigital cells is a well-known example of programmed cell death during development of the mouse embryo^42^, and is considered to be mainly mediated by apoptotic^28^ and caspase-dependent cell death^29^,^30^. However, in addition to AO-positive apoptotic cells, we unexpectedly found a large number of PI-positive necrotic cells in the interdigital region (Fig. 1). Although these PI-positive cells had different morphological features from typical apoptotic cells (Fig. 1f,g), PI foci were almost completely absent in *Bax*/*Bak* KO mice and *caspase-9* KO mice, both of which show deficiency of apoptosis (Fig. 2). These observations suggested that the necrotic cells in the interdigital regions represented apoptotic cells undergoing secondary necrosis. According to many previous reports, apoptotic cells are rapidly engulfed by phagocytes *in vivo*, but this might not be the case during embryogenesis. In some tissues such as the interdigital regions, so many cells die by apoptosis during embryogenesis that phagocytes might not be able to process all of the apoptotic cells rapidly enough, leaving many cells to undergo secondary necrosis. Because very few PI-positive cells were also AO-positive (Fig. 1c), necrotic cells might be removed by different mechanisms from those acting on apoptotic cells.

In addition, our *in vivo* staining method for necrosis revealed the occurrence of intrinsic necrotic cell death at the prospective bone surface, and this form of cell death did not occur in mice with deficiency of the autophagy-related gene *Atg9a*. We found that the bone surface was rougher and more porous in newborn *Atg9a* KO mice than in wild-type mice, which supports the notion that Atg9a-dependent necrotic programmed cell death has biological significance. The role of Atg9a in necrotic programmed cell death raised the possibility of autophagic death being involved in this process. However, this form of necrotic death was found to occur in embryos with deficiency of *Atg5*, another autophagy-related gene (Fig. 4c,d), and autophagosomes were not observed in the dead cells (Fig. 3e,f), suggesting that autophagy does not have a crucial role in the necrotic programmed cell death at the prospective bone surface. Our results also indicated that Atg9a-dependent necrosis is distinct from necroptosis, based on our findings in RIP1-deficient mice and IHC using an antibody for a marker of necroptosis (Fig. 5). Furthermore, the morphological features of the necrotic cells at the prospective bone surface differed from those previously reported for cells undergoing necrotic programmed death *in vivo*, such as linker cell death^3^, or cell death during mammary gland regression^3^,^4^. Atg9a is known to have a role in inflammation^36^, but its precise role in necrotic programmed cell death at the prospective bone surface in embryonic mice needs to be elucidated in the future.

The necrotic cells at the prospective bone surface were surrounded by collagen fibrils and the lamina limitans (organic material between the mineralized and non-mineralized parts of the bone matrix) was adjacent to these cells (Fig. 3e,f), raising the possibility that the necrotic cells triggered bone mineralization and subsequent calcification. It is thought that calcification of bone is initiated from small extracellular vesicles, referred to as ‘matrix vesicles'^43^,^44^, in which crystals of calcium phosphate form and grow until they break through the vesicle membrane. Protruding calcium phosphate crystals then assemble radially and form spherical mineralized structures, which are referred to as ‘mineralized nodules'. This process is called ‘matrix vesicle mineralization'^45^. The most influential hypothesis is that matrix vesicles arise by budding from the plasma membrane of chondrocytes^46^, but there have also been some reports that degenerating cells act as sites for initiation of calcification like matrix vesicles^47^,^48^. The necrotic cells associated with the lamina limitans that we observed at the prospective bone surface were in a similar situation to the previously reported degenerating cells associated with mineralization. Because we observed malformation of the bone surface in *Atg9a* KO mice, it seems that Atg9a-dependent necrosis has an important role in bone mineralization/calcification. Further investigations are needed to determine whether Atg9a has a direct role in the cell death pathway and whether cell death directly induces bone calcification.

## Methods

### Mice

The *Atg5*, *Bax*/*Bak*, *caspase-9* and *RIP1* KO mice were provided from Dr N. Mizushima (Tokyo Medical and Dental University [TMDU]), Dr C. Thompson (Memorial Sloan Kettering Cancer Center), Dr K. Kuida (Millennium Pharmaceuticals, Inc.) and Drs. M.A. Kelliher (University of Massachusetts Medical School) and J. Silke (Walter and Eliza Hall Institute), respectively. *Atg9a* KO mice were previously described^36^. Wild-type C57BL/6 J and 129X1/Sv mice were purchased from Japan SLC, Inc. Apart from *Atg9a* KO mice, the KO mice were backcrossed to a C57BL/6 J background for at least 10 generations. However, *Atg9a* KO mice had a mixed C57BL/6 J and 129X1/Sv background because mice with a pure C57BL/6 J background died before E14.5. E0.5 was designated as noon on the day when a copulation plug was identified. All mice were reared under specific pathogen-free conditions. Experiments were performed according to the protocol approved by the Ethics Review Committee for Animal Experimentation of Osaka University Graduate School of Medicine and the Institutional Animal Care and Use Committee of Osaka Medical Center for Cancer and Cardiovascular Diseases.

### Vital staining

Embryos were dissected from the uterus and placed in PBS(−) at 37 °C. Then each embryo was placed in embryo culture medium (Dulbecco's modified eagle's medium (DMEM) with 10% FBS and 25 mM HEPES) at 37 °C. While the embryos were in the culture medium, 1 μg of AO (Sigma) and 0.5 μg of PI (Dojindo) in 1 μl of PBS (−) were injected into the yolk sac vein. After incubation was performed for 30–60 min, the embryos were dissected on ice-cold PBS (−). For TEM analysis, 1 μg of AO and 0.5 μg of PI in 1 μl of PBS (−) were injected into the yolk sac vein of embryos, which were still connected to their mother. After 30 min, the embryos were harvested and then dissected in ice-cold PBS (−).

### TUNEL staining and IHC

Unfixed samples were directly embedded in optimal cutting temperature (OCT) compound (Tissue-Tek) and frozen with dry ice. The frozen samples were cut into 6 μm sections using a cryomicrotome and the sections were fixed with 4% paraformaldehyde (PFA). TUNEL staining was carried out according to the manufacturer's protocol in the ‘ApopTag Peroxidase *In Situ* Apoptosis Detection Kit' manual (S7100: Chemicon). For detection of macrophages, monoclonal anti-F4/80 antibody (sc-59171: Santa Cruz) was applied to the sections at a dilution of 1:50. Signals were detected by Simple Stain Mouse MAX PO (Rat) (Nichirei) and visualized using ImmPACT DAB (Vector Laboratories). For detection of cleaved caspase-3, LC3 and phospho-MLKL, monoclonal anti-cleaved caspase-3 (Asp175) antibody (#9664: Cell Signaling) at a dilution of 1:200, polyclonal anti-LC3 antibody (PM036: MBL) at a dilution of 1:10,000, or monoclonal anti-phospho MLKL (Ser345) antibody (ab196436: Abcam) at 0.5 μg ml^−1^ were respectively applied to the sections. Then signals were detected with Signal Stain Boost IHC Detection Reagent (HRP, Rabbit) (Cell Signaling) and visualized using ImmPACT DAB.

### Transmission electron microscopy

Tissues harvested from embryos injected with AO and PI were fixed for 2 h at room temperature in 0.1 M phosphate buffer (pH 7.3) containing 1.5% PFA and 3% glutaraldehyde, followed by cryoprotection with 30% sucrose. Then the tissues were embedded in OCT compound, frozen in nitrogen slush, and cut into 8–10 μm sections. The cryosections were first observed with a fluorescence microscope, and then were fixed for 30 min at room temperature in 0.1 M phosphate buffer (pH 7.3) containing 1.5% PFA and 3% glutaraldehyde. They were further fixed for 30 min at 4 °C in 0.1 M phosphate buffer (pH7.3) containing 1% osmium tetroxide. After embedding in Epon812 (TAAB), semi-thin sections were cut and stained with toluidine blue. The tonal range of the fluorescence micrographs was adjusted and these micrographs were merged with the toluidine blue-stained semi-thin sections to compensate for the reduced fluorescence intensity of the fixed and cryoprotected samples. Then ultra-thin sections (∼90 nm) were cut, stained with aqueous lead citrate and uranyl acetate and examined with an H7650 electron microscope (Hitachi) at an acceleration voltage of 80 kV or with an H7100 electron microscope (Hitachi) at an acceleration voltage of 75 kV. To obtain the electron micrographs presented in Fig. 1f, 16 images were arranged and then adjustment of image contrast was performed. Tissues obtained from embryos without injection of AO and PI were fixed overnight at 4 °C in 0.1 M phosphate buffer (pH 7.3) containing 1.5% PFA and 3% glutaraldehyde, and fixed for 1 hr at 4 °C in 0.1 M phosphate buffer (pH7.3) containing 1% osmium tetroxide. After embedding in Epon812, ultra-thin sections (∼90 nm) were cut, stained with aqueous lead citrate and uranyl acetate, and examined with an H7100 electron microscope at an acceleration voltage of 75 kV.

### Dahl's calcium staining

Unfixed samples were directly embedded in OCT compound, frozen in dry ice, and cut into 6 μm sections on a cryomicrotome. The sections were fixed in 99.5% ethanol, stained for 5 min with 1% Alizarin Red S (Sigma) (pH=6.3), and then washed with water.

### Alcian blue and alizarin red staining of cartilage and bone

Heterozygous *Atg9a* mice and heterozygous *Atg5* mice were crossed with respective siblings. Newborn pups were killed, skinned, eviscerated and fixed in 95% ethanol for 1 day, followed by defatting with acetone for 1 day. Samples were placed in Alcian Blue solution (0.015% Alcian Blue 8GX (Sigma) in 80% ethanol/20% glacial acetic acid) for 24 h to stain cartilage, followed by two washes in 95% ethanol (for 24 h each) and clearing in 1% KOH for 5 h before overnight incubation in Alizarin Red solution (0.005% Alizarin Red S in 1% KOH) to stain bone. Then the samples were soaked in 1% KOH/20% glycerol for 2 days for clarification, and placed in a 1:1 mixture of glycerol and ethanol for storage. After removing the right forelimbs and hind limbs, the transparent specimens were photographed on a light box using a digital camera. The removed forelimbs were examined by laser scanning confocal microscopy using Alizarin Red S fluorescence. Three-dimensional-rendering of z-stack images was done using Volocity (PerkinElmer), and the intensity of Alizarin Red S staining was measured by Fiji^49^. Areas with an intensity of <10% were defined as cavities, and the ratio of the cavity area to the total area was calculated.

### Statistical analyses

The data represent the mean±s.d. The biological repeats are indicated by ‘n' in the figure legends. Data were analysed by the two-tailed unpaired Student's *t*-test. *P* values of <0.05 were considered to be significant. Sample size was not estimated. The experiments were repeated until at least three control and knockout embryos/pups were obtained. The parents of mice used in the experiments were randomly chosen from our in-house colonies. Litters from the parents were not excluded from the analysis. Experiments of KO embryos or newborn mice were performed before confirmation of these genotypes.

### Data availability

No data sets were generated or analysed during the current study. Therefore, the authors declare that all data supporting the findings of this study are available within the article and its supplementary information files or from the corresponding author upon reasonable request.

## Additional information

**How to cite this article:** Imagawa, Y. *et al*. Vital staining for cell death identifies Atg9a-dependent necrosis in developmental bone formation in mouse. *Nat. Commun.* **7,** 13391 doi: 10.1038/ncomms13391 (2016).

**Publisher's note:** Springer Nature remains neutral with regard to jurisdictional claims in published maps and institutional affiliations.

## Supplementary Material

Supplementary Information Supplementary Figures 1-5
