# Supplementary material for: Vital staining for cell death identifies Atg9a-dependent necrosis in developmental bone formation in mouse
Source: Nat Commun. 2016 Nov 4;7:13391. doi: 10.1038/ncomms13391 (PMC5097171; doi:10.1038/ncomms13391)
Supplement: Supplementary Information — Supplementary Figures 1-5 [file ncomms13391-s1.pdf]

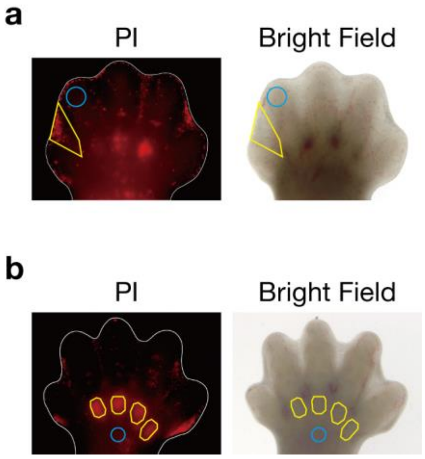

Supplementary Figure 1: PI foci in the first interdigital region and the phalangeal area

(a) Fluorescence stereomicroscopy of the right forelimb bud of an E13.5 embryo injected with AO and PI. The yellow quadrilateral indicates the first interdigital region, and the cyan circle indicates the second digit (background area). The relative intensity of PI signals in the first interdigital region was calculated as the mean value of PI signals in the interdigital region divided by that in the second digit. (b) Fluorescence stereomicroscopy of the right hind limb bud of an E14.5 embryo injected with AO and PI. Four yellow hexagons indicate the PI-positive phalangeal area, and a cyan circle indicates palmar region (background area). The relative intensity of PI signals in the phalangeal area was calculated as the mean value of PI signals in the area divided by the mean value in the palmar region.

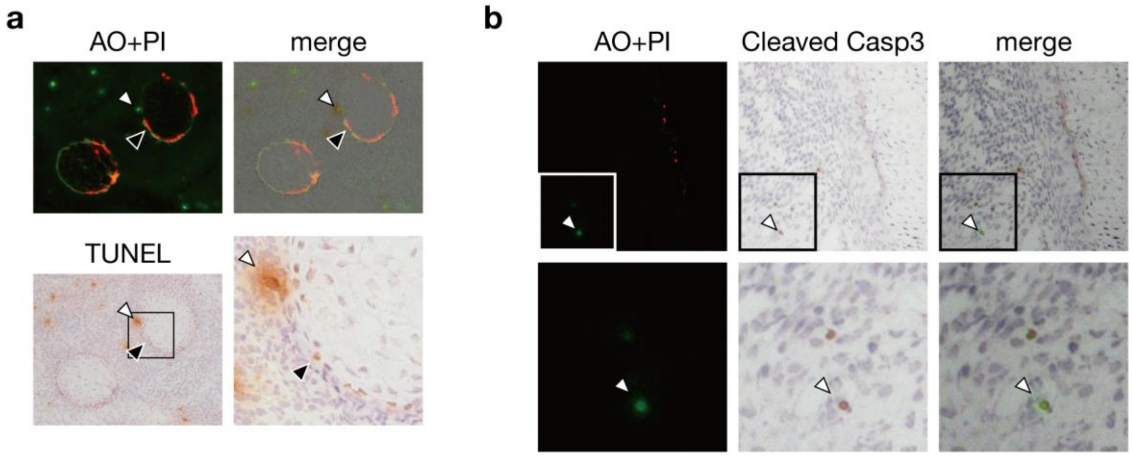

Supplementary Figure 2: TUNEL staining and IHC for cleaved caspase-3 of AO-positive cells and PI-positive cells

(a) PI-positive cells show weaker TUNEL staining than AO-positive cells. A transverse cryosection of the forelimb of an E14.5 embryo injected with AO and PI was observed by fluorescence microscopy, followed by TUNEL staining. White and black arrowheads indicate AO and PI foci, respectively. (b) An AO-positive cell is also positive for cleaved caspase-3 in the same section as that shown in Fig. 3d. A vertical cryosection of the forelimb of an E14.5 embryo injected with AO and PI was subjected to immunostaining by an anti-cleaved caspase-3 antibody. White arrowhead indicates the AO focus.

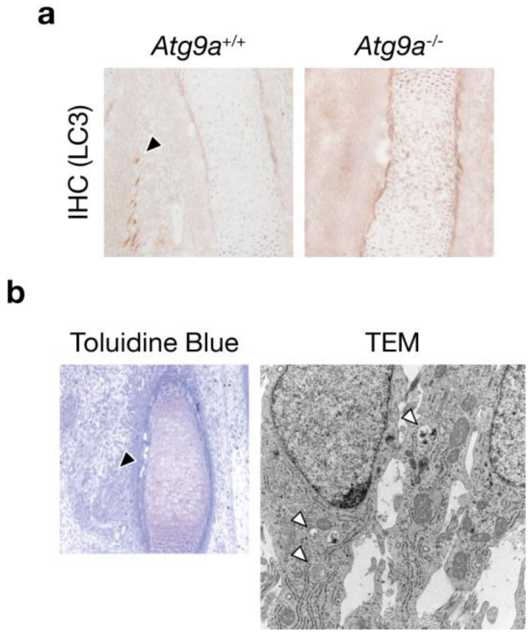

Supplementary Figure 3: Detection of autophagy

(a) Induction of LC3 and punctae formation in the same section as that shown in Fig. 4e. Vertical cryosections of the forelimb of an E14.5 *Atg9a* KO (*Atg9a*$^{-/-}$) embryo and a control (*Atg9a*$^{+/+}$) embryo injected with AO and PI were subjected to immunostaining by an anti-LC3 antibody. Black arrowhead indicates one of the aligned LC3 foci. No induction of LC3 and punctae formation in *Atg9a* KO embryos. (b) Autophagosomes in the same area as the LC3 foci. Black arrowhead indicates the site observed by TEM. White arrowheads indicate autophagosomes.

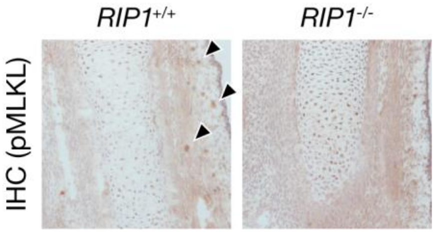

Supplementary Figure 4: Detection of necroptosis
Phosphorylation of MLKL observed in the same section as that shown in Fig. 5b. Vertical cryosections of the forelimb of an E14.5 *RIP1* KO (*RIP1*$^{-/-}$) embryo and a control (*RIP1*$^{+/+}$) embryo injected with AO and PI were subjected to immunostaining by an anti-phospho MLKL antibody. Black arrowheads indicate phospho-MLKL foci. There is no phosphorylation of MLKL in the *RIP1* KO embryo.

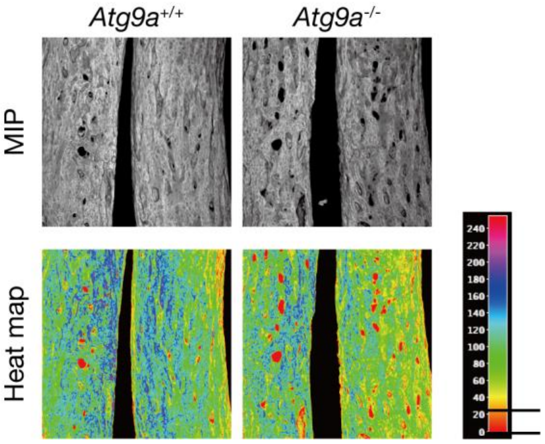

Supplementary Figure 5: Analysis of the cavity area in forelimb zeugopods
Transparent specimens of *Atg9a* KO (*Atg9a*$^{-/-}$) and control (*Atg9a*$^{+/+}$) newborn pups were stained by Alcian Blue and Alizarin Red S. Forelimb zeugopods were examined by laser scanning confocal microscopy using Alizarin Red S fluorescence, after which maximum intensity projection (MIP) images were constructed (upper panels) and the intensity of Alizarin Red S staining was shown as heat map (lower panels). Color legend of the heat map is shown on the lower right. Areas with an intensity < 10% were defined as cavities, and the ratio of the cavity area to the total area was calculated.
